# Sex differences in the outcomes of modifiable lifestyle factors for cognitive aging: neuroinflammation and microglia as key underlying mechanisms

**DOI:** 10.3389/fnagi.2025.1642043

**Published:** 2025-07-22

**Authors:** Samantha G. Coleborn, Zoë M. Gilson, Yunyong Guo, Marie-Ève Tremblay

**Affiliations:** ^1^Institute on Aging and Lifelong Health, University of Victoria, Victoria, BC, Canada; ^2^Department of Psychology, University of Victoria, Victoria, BC, Canada; ^3^Department of Computer Science, University of Victoria, Victoria, BC, Canada; ^4^School of Medical Sciences, University of Victoria, Victoria, BC, Canada

**Keywords:** microglia, cognitive aging, sex differences, diet, exercise, social isolation, animal models, human studies

## Abstract

Microglia are the resident immune cells of the brain. Over the past two decades, they have been shown to play critical roles throughout life. Microglia are now considered to be important for brain formation, maturation, activity and plasticity, with outcomes on behavior and other cognitive domains. With this knowledge, microglia represent a promising therapeutic target to promote brain health along an aging trajectory. Emerging evidence also indicates that modifiable lifestyle factors for cognitive aging can influence the brain and behavior by acting on microglia. The mechanisms identified so far involve their roles in synaptic plasticity, axonal myelination, and adult neurogenesis, exerted through the modulation of brain inflammation (‘neuroinflammation’), the release of trophic factors, and phagocytosis. In this mini-review, we will cover the outcomes of exercise, diet. and social isolation on microglial functions during aging. Sex differences in the identified outcomes on cognitive aging and the underlying mechanisms will be highlighted. Our goal with this mini-review is to stimulate further research on this important topic.

## Introduction

Microglia are the immune cells of the central nervous system (CNS) that can clear toxic debris, remove dysfunctional or degenerating cells, as well as pathogens, modulate inflammation, and play many other roles (Paolicelli et al., 2022). Contrary to blood immune cells, microglia originate from the embryonic yolk sac (Paolicelli et al., 2022; Saijo and Glass, 2011). They enter the CNS during early development, and they stay there throughout life, maintaining their numbers through self-renewal (Paolicelli et al., 2022; Tay et al., 2017).

In 2005, it was first discovered, using minimally invasive two-photon *in vivo* imaging, that microglia, which used to be called ‘resting’ or ‘quiescent’ in the healthy CNS, are instead extremely dynamic, continuously surveying the parenchyma with their highly motile processes (Paolicelli et al., 2022; Tremblay et al., 2011). This seminal discovery has stimulated an exponential growth of microglial research about the roles of homeostatic microglia, which are exerted through their bi-directional interactions with other CNS cells, and about the underlying molecular mechanisms (Tremblay et al., 2015). Studies in the field using various novel tools have increasingly revealed that microglia are crucial for CNS formation, maturation, activity, plasticity, and integrity, as well as behavior and cognition, across the lifespan (Paolicelli et al., 2022; Šimončičová et al., 2022). Microglial roles involve their regulation of neurogenesis, vascular remodeling, blood–brain barrier, astrocyte and oligodendrocyte modulation, synapse remodeling, neuromodulation and the immune response (Šimončičová et al., 2022). More recently, microglia were also shown to contribute to the process of axonal myelination (Sierra et al., 2024). These various microglial roles are exerted via their dynamic surveillance of the CNS parenchyma, release of soluble factors such as inflammatory mediators, cell–cell interactions, and phagocytosis throughout life (Paolicelli et al., 2022; Sierra et al., 2024).

However, these beneficial roles were shown to become compromised with exposure to environmental risk factors for disease, such as stress and aging, combined with genetic vulnerabilities, leading to cognitive decline and neurodegenerative diseases along an aging trajectory (Depp et al., 2025). Recently, various microglial states that perform different, often altered functions were also discovered, and their unique molecular signatures have started to unravel, providing novel opportunities for therapeutic intervention (Paolicelli et al., 2022; Depp et al., 2025; Stratoulias et al., 2019). Among these states associated with aging, microglia were shown to become dystrophic and senescent, in which their beneficial roles are impaired (Stratoulias et al., 2019; Carr et al., 2025). The targeting of microglial states was thus proposed to represent the future of neurological health (Šimončičová et al., 2022). Pharmacological treatment strategies acting on microglia and their states are currently under clinical trials (Šimončičová et al., 2022).

In addition, microglia have emerged as highly responsive to several modifiable lifestyle factors known to impact cognitive function, such as exercise, diet, and social interactions (Madore et al., 2020; Jiao et al., 2024; Augusto-Oliveira and Verkhratsky, 2021). These modifiable factors were suggested to prevent the transition of homeostatic microglia to dystrophic and senescent microglial states, protecting against cognitive decline during aging (Augusto-Oliveira and Verkhratsky, 2021; Duggan and Parikh, 2021). In this mini-review, we discuss the outcomes of exercise, diet and social isolation on microglial functions during aging. Sex differences in the identified outcomes on cognitive aging and their mechanisms are also highlighted. Our goal with this mini-review is to stimulate further research on this important topic to pave the way toward the design of multi-modal interventions that promote microglial health.

## Modifiable lifestyle factors

### Exercise

Exercise confers beneficial effects on cognitive aging (Colcombe and Kramer, 2003; Mandolesi et al., 2018; Xu et al., 2023). While the precise cellular mechanisms underlying its effects remain unknown, there is current research to suggest exercise may exert its beneficial influence through modulating inflammatory and immune responses as well as microglial activity (Duggan and Parikh, 2021; Strohm and Majewska, 2024; Vukovic et al., 2012). As indicated in previous reviews, exercise has been shown to modulate microglial functions across various animal models in both healthy and disease states (Strohm and Majewska, 2024). Utilizing rodent models of the healthy aging brain, exercise has been found to impact microglia density, phenotype, and functionality across neural regions, particularly in the hippocampus suggesting microglia serve an important role in the relationship between exercise and brain health (Strohm and Majewska, 2024; Sun et al., 2017). For example, a study by Vukovic et al. (2012) demonstrated that hippocampal microglia influence the impact of exercise on adult neurogenesis in adult female *Csf1r-GFP* transgenic mice.

Previous reviews have proposed that exercise’s influence on microglial activity may be due to its known impacts on microglial microenvironment by altering signaling molecules such as neurotrophic factors (brain derived growth factor (BDNF), insulin-like growth factor (IGF), and nerve growth factor) and neurotransmitters (norepinephrine, serotonin, and dopamine) (Strohm and Majewska, 2024).

Meta-analyses of random control trials in older adults have indicated that there exist sex differences in exercise’s influence on cognition (Barha et al., 2017), yet most studies have exclusively investigated the cognitive outcomes following exercise in male populations (Barad et al., 2023; Cortes and De Miguel, 2022). Moreover, despite microglia in both rodent models and humans having sex-dependent characteristics, the literature has failed to explore whether the impact of exercise on microglia varies between the sexes (Strohm and Majewska, 2024; Barad et al., 2023).

Following our literature search across databases (PubMed, Psycinfo, Medline), only one study emerged that explicitly explored whether exercise impacts microglia in a sex-dependent manner (Kohman et al., 2013). This study found that sex mediates the relationship between voluntary wheel running and microglia activity in the hippocampus of adult (4 months) and aged (21–22 months) male and female BALB/c mice. Specifically, aged female mice within the exercise condition showed a decrease in both microglia expressing cluster of differentiation (CD)86, and major-histocompatibility complex (MHC) II relative to female aged controls, while aged male mice instead demonstrated a decrease in microglia expressing CD86 and an increase in MHC II compared to their respective control group (Kohman et al., 2013). The upregulation of CD86 and MHC-II indicate increased microglial reactivity, with the former being a co-stimulatory lysosomal molecule expressed by microglia when they are in active state, and the latter being cell surface proteins whose expression is integral for immune responses made by microglia of a variety of phenotypes (Tremblay and Verkhratsky, 2024). These results thus suggest the impact of exercise on microglial activity is not only dependent on age but also on sex.

Following this search, no study to date has investigated whether there are sex-specific cognitive effects following microglial regulation from an exercise intervention, demonstrating a crucial gap in the literature given the known sex-differences in cognitive aging, microglial functioning, and response to exercise.

### Diet

Diet is an important lifestyle factor known to impact cognitive health (Cao et al., 2016; Martínez-Lapiscina et al., 2013; Morris et al., 2015; Solfrizzi et al., 2003). Diet also exerts significant influences on microglia function and phenotype (Duggan and Parikh, 2021; Spencer et al., 2019). For example, Baufeld et al. (2016) demonstrated that a high fat diet (HFD) increases hypothalamic microglia in adult male C57BL/6J mice after 8 weeks; in obese humans, they observed dystrophic changes to microglia structure that correlated with body mass index. There is growing evidence to suggest microglia are important contributors to the relationship between diet and cognitive aging (Duggan and Parikh, 2021; Spencer et al., 2019; Ledreux et al., 2016). Studies demonstrate that young adult F344xBN F1 rats placed on a HFD display changes to microglial phagocytic activity within the amygdala, and such changes are linked to deficits in amygdala-dependent memory tasks (Spencer et al., 2019). Moreover, HFDs have also been associated with increased microglial cell bodies, processes, and staining (as determined by OX-6, an antibody targeting MHC II thus indicating the presence of microglial reactivity) in the hippocampus for both young and aged rodents relative to young rodents on a control diet, with aged rodents also demonstrating significant memory impairments following these changes to microglial characteristics (Tremblay and Verkhratsky, 2024; Ledreux et al., 2016).

Some studies have investigated whether the impact of diet on microglia and cognitive functioning in aged animal models varies between the sexes. Some studies (Abi-Ghanem et al., 2023; Gannon et al., 2022) have specifically employed dementia mouse models to explore how diet impacts microglial activity and morphology to influence the cognitive outcomes and neuropathology of various dementias.

Abi-Ghanem et al. (2023) utilized middle-aged C57BL/6J mice placed on either a HFD or control diet and subjected to a vascular cognitive impairment (VCID) or sham surgery to explore potential sex differences between diet-induced microglial changes in VCID. The morphology and immunoreactivity of microglia were measured through the presence of ionized calcium adapter-binding molecule (Iba)1, which permits the identification of microglia regardless of activity state, and CD68, indicating microglial phagolysosomal activity. The authors found that in the VCID model, a HFD was associated with worse metabolic and cognitive outcomes for females rodents, and there were sex-differences regarding how a HFD influences microglial density and activity in the cornu ammonia (CA)1 region of the hippocampus, a neural structure important for episodic memory, and corpus callosum (CC), which transmits information between the brain’s hemispheres. Specifically, a HFD was associated with an increase in ‘phagocytic’ microglia in males and decrease in females within the CC. Furthermore, an increase in phagocytic microglia was found within the CA1 region of the hippocampus for males but not females. Regardless of sex, increased phagocytic microglial activity within the hippocampus, but not the CC, was linked to worse episodic memory performance. Thus, the authors concluded there is a sex-dependent effect of a HFD on cognitive impairment, where phagocytic microglial activity may be an underlying mechanism for this relationship in males but not females.

Gannon et al. (2022) also sought to explore whether the influence of HFD on cognitive outcomes in specific dementia mouse models is sex dependent. Utilizing adult wild-type (WT) B6129SF2/J (healthy control) and 3×Tg-AD mice [animal model of Alzheimer’s disease (AD)] pathology that underwent either a sham or unilateral common carotid artery occlusion surgery, the authors explored the impact of diet in AD or mixed dementia (MxD) models on cognitive outcomes and neuropathology such as neuroinflammation. ‘Microgliosis’ measured by Iba1 and CD68 presence served as a marker of neuroinflammation in this study. The authors determined that the metabolic effects of a HFD were more pronounced in females, and that AD and MxD females fed a HFD showed greater impairments in cognition and activities of daily living. Regarding sex differences in microgliosis, greater glucose intolerance was associated with increased Iba1 and CD68 presence within the CA1 and CA2 regions of the hippocampus (neural structures associated with episodic and social memory) in female but not male mice. The authors suggest such findings indicate a sex-dependent association between microgliosis and metabolic markers and stress that these results demonstrate a need to address how diet contributes to sex-specific differences in dementia.

In addition to specific dementia animal models, there have also been research efforts exploring sex differences in the impact of diet on cognitive outcomes in the context of normal aging. Evans et al. (2024) explored the influence of a HFD on behavior and neurodegeneration and how these associations differ between male and female C57BL/6J mice. This study found that a HFD exerts a sex-dependent effect on learning and memory in male but not female mice, where specifically a HFD negatively impacted spatial learning and recall in young male mice, and cued memory recall in both young (2–3 months) and older (12–13 months) male mice. Moreover, a HFD was found to potentiate microglial immunoreactivity (i.e., an increased number of cells expressing Iba1 and CD68) within the CA1 hippocampal region of male mice, providing possible mechanisms into how diet impacts cognitive functioning for male populations.

One final paper exploring the sex-dependent cognitive effects of HFDs investigated how HFD impacts the regulatory role of microglia on adult hippocampal neurogenesis (AHN) and subsequent outcomes on cognitive aging. This study conducted by Robison et al. (2020) determined that the effect of HFD on AHN in C57BL/6 J mice differs between the sexes (where HFD only decreased AHN in females), and males fed a HFD demonstrated a significantly greater number of microglia (Iba1 and CD68 positive) within the inner and outer areas of the dentate gyrus, whole hippocampus, and dorsal and ventral subregions. The authors suggest that the increased presence of microglia in males was protective of AHN and hypothesized females have a sex-specific microglia impairment in the presence of an HFD.

### Social isolation

Experiences of social isolation throughout the lifespan have impacts on microglial density and function. Early-life social isolation and maternal separation studied in young DBA/1J mice has been shown to reduce microglial volume in the hippocampus, resulting in an increase of depressive behaviors (Gong et al., 2018). Another study by Al Omran et al. (2022) found that chronic social isolation in adult C57BL/6 mice, induced over a period of 4 weeks, showed a decrease in number and reactivity (determined by significant morphological changes including decreases in lacunarity, perimeter, and cell size, and increases in cell density) of microglia marked with Iba1 and CD11b in the dentate gyrus of the hippocampus. Social isolation is also associated with poor cognitive outcomes in aging human subjects in many behavioral studies (Evans et al., 2019; Guarnera et al., 2023), but the role of microglia and neuroinflammation as an underlying mechanism in this relationship has yet to be determined.

Our literature search revealed only one study that examined the sex-specific effects of social isolation on microglia (Vu et al., 2023). The researchers found that social isolation resulted in significantly increased microglial volume in the dorsomedial hippocampus and hypothalamus in male compared to female C57BL/6 mice. Following a two-week period of isolation, there were significant increases in microglial branching, territory occupancy, and end points in the dorsomedial hypothalamus and hippocampal CA2 region of male mice as measured by Iba1 and CD86, where females showed this effect in the hypothalamus only. Males also showed decreases in the microglial marker CD68 in these regions, where females showed an increase in CD68 in the hypothalamus only. Notably, while the researchers did include a social interaction task following isolation, there was no cognitive outcome measure included. Therefore, even though the results show that microglia are impacted by social isolation, we cannot conclude that microglia are involved in the mechanisms behind social isolation and cognitive performance.

It is important to note that this study explored this sex-specific effect in adult mice only, and not aged mice, and therefore cannot be used to draw conclusions about this sex-specific effect on cognitive aging. Overall, there is minimal research looking at sex differences in the impacts of social isolation on microglia, and no research investigating how microglia respond to isolation in aging subjects.

## Discussion

**Figure 1:**
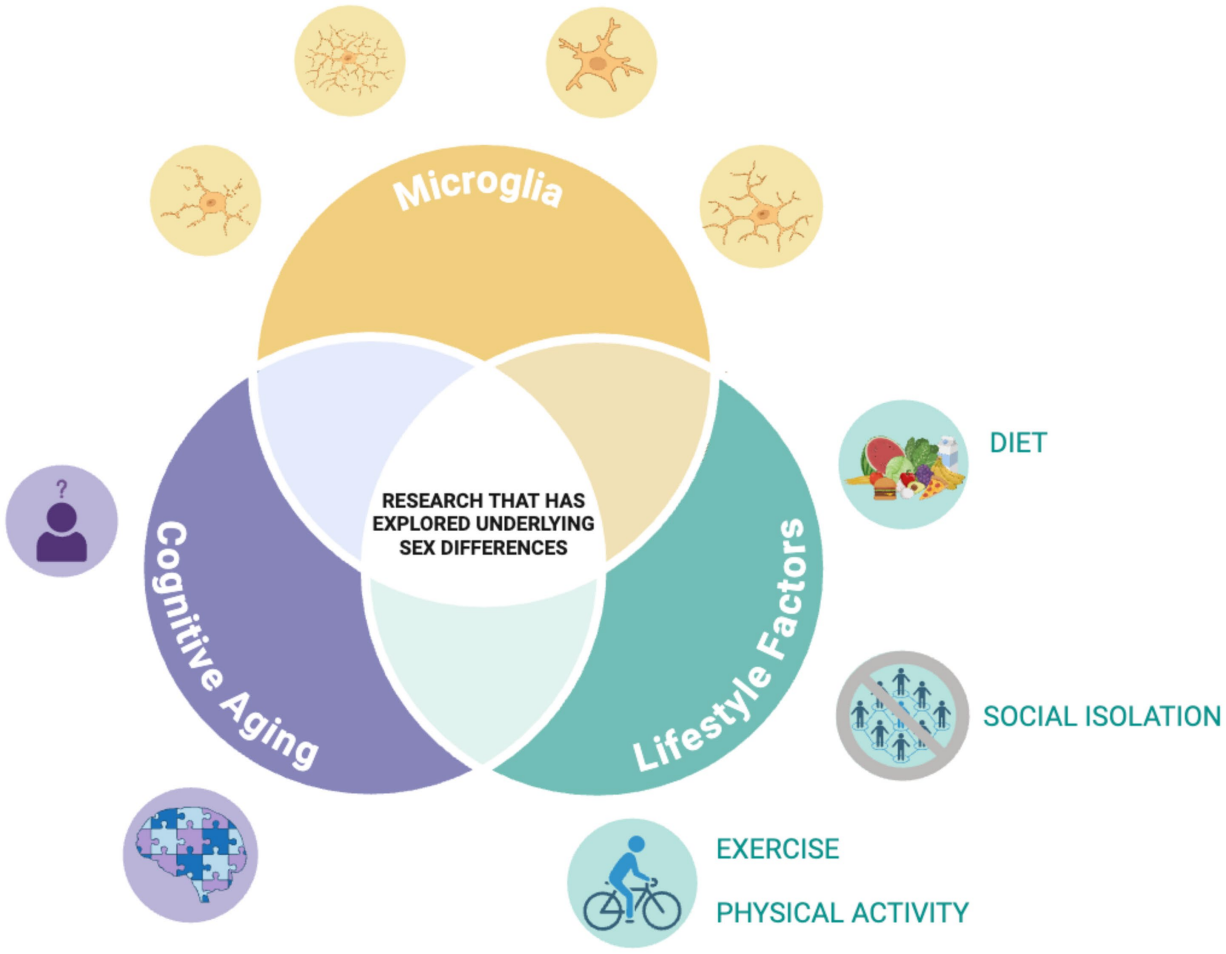
Illustration of identified literature gap. Illustration depicting the research gap our team is highlighting. Through our review, although not a scoping or systematic review, we found there is little research to date that has explored how the relationship of the lifestyle factors with microglia structure/function during aging varies across the sexes. Figure was created with BioRender.

This mini-review highlights the complex interplay between modifiable lifestyle factors, microglial activity, and cognitive aging, with an emphasis on sex differences. While the evidence is still emerging, several consistent patterns and critical gaps have been identified, which need further study. Figure 1 illustrates the lack of research exploring these factors comprehensively.

From our review, there was the most literature showing sex specific effects in diet studies. For instance, three out of the four studies in this review indicated that there is an increase in microglial density and change in function for male populations, whereas females showed either no change or a different pattern of microglial response. These findings suggest that males may be more susceptible to the negative cognitive impacts of HFDs. Exercise was also consistently shown to modulate microglial activity, but the sex-specific effects of exercise on microglia and cognition are not well understood, notably due to a bias toward male studies in the literature and a lack of studies investigating sex as a biological variable. Exercise can exert sex-dependent effects on microglial activity, but more work needs to be done to confirm these findings and explore their implications for cognition across sexes. Additionally, it would be helpful to compare exercise regimens to explore sex-dependent effects in future studies.

Alongside these consistent findings, there were still many discrepancies across studies examining sex differences in the impacts of modifiable lifestyle factors on microglia. Some of these discrepancies may be due to differences in the markers that were used, the brain regions that were examined in these studies, or differences in animal strains and ages. While the majority of the literature on HFD in this review documented increased microglial changes in males, Robison et al. (2020) reported that increased microglial presence in males on a HFD was neuroprotective against AHN, while females exhibited impairment. This is an indication of the need to explore further why microglial state transformation in HFD can be detrimental or protective in males and why this could be different in females. The few studies on social isolation and microglial activation show conflicting results. Vu et al. (2023) show that male mice exhibited greater microglial volume and branching with social isolation than females. The absence of cognitive outcome measures in this study cannot provide conclusions about the probable effects of microglia in mediating the cognitive effects of social isolation.

There is a significant gap in understanding the sex-specific effects of exercise on microglial activity and cognitive outcomes. The role of microglia in mediating the cognitive effects of social isolation, particularly in aging populations, remains largely unexplored. The majority of the reviewed studies were conducted in animal models, limiting the generalizability of the findings to humans. Future research should aim to translate these findings to human populations. To this end, it will be important to develop microglial state-specific radiotracers that allow to track transformations in their population over time (Tremblay, 2025), notably in response to modifiable lifestyle changes.

## Conclusion

This mini-review serves to demonstrate the importance of consideration of sex differences in examining the impact of modifiable lifestyle factors on cognitive aging and the microglial contribution to this. While the impact has been discovered concerning the influence of diet, exercise, and social isolation on microglial activity, critical gaps remain, including an important need for clarification of sex-specific effects and translation of findings from animal models to human populations.

The converging evidence on the sex-dependent effects of diet on microglial function and cognitive outcomes highlights the importance of biologically sex-based personalized interventions. Similarly, the lack of information on exercise and social isolation suggests that these are areas which need further investigation, particularly in the context of aging, and across sexes.
